# Frequency of Repeating Antinuclear Antibody Testing: When Less Is More

**DOI:** 10.7759/cureus.52347

**Published:** 2024-01-15

**Authors:** Mahadi B Alyami, Mohammed N Hakeem, Abdulaziz I Fadil, Bassim A Jee, Hamza M ElAbbasy, Ghada Ankawi

**Affiliations:** 1 Medicine, King Abdulaziz University Faculty of Medicine, Jeddah, SAU

**Keywords:** laboratory investigations, rheumatology, connective tissue diseases, ana profile, antinuclear antibody

## Abstract

Objectives

Antinuclear antibodies (ANA) are autoantibodies that are associated with and ordered to diagnose autoimmune connective tissue disease. ANA have high sensitivity (~98%) but low specificity (~75%), and because they can be found in healthy individuals and non-rheumatologic conditions leading to their elevation, ANA tests are often requested and interpreted inappropriately by clinicians. The aim of this study was to retrospectively assess how frequently ANA testing is repeated in the adult population of Saudi Arabia (SA) and which factors are associated with and lead to inappropriate testing.

Methodology

We investigated a study group of 40,634 adult patients who underwent 229,825 ANA tests from 2018 to 2022 in an academic hospital in Jeddah, SA. We took a random sample of 500 patients from the study group, along with their 998 ANA tests, to look in depth into our research questions. Variables related to patients, ANA tests, and ordering physicians were collected. Descriptive and analytical statistics were employed to address the research questions, and a p-value < 0.05 was considered statistically significant.

Results

We found 57% of the ordered ANA tests to have positive results, with the most common titers of mild positivity being 1:80 and 1:160. Most repeated ANA tests were ordered with an interval of more than one year, and when repeated, 67% of test results remained unchanged. The majority of seroconversions resulted from negative ANA tests or those with weak (titer 1:40) or mild positivity (titer 1:80-1:160). The results of the moderate (titer 1:320-1:640) and strong (titer ≥1280) positivity ANA tests did not change. Only 11% of repeated ANA tests were found to be appropriate for repetition. The most common specialties associated with ordering ANA tests in general were internal medicine, followed by rheumatology, and finally family medicine. Our correlation analysis revealed that being female, having systemic connective tissue disease, and having a rheumatologist as a specialist were all associated with ordering more than 10 ANA tests (p < 0.05).

Conclusion

Because the results of repeated ANA tests did not change much, our study suggests that the cost of repeating ANA tests and the subsequent potentially unnecessary interventions should all be carefully examined before scheduling a repeated ANA test. Further studies involving patients from SA and across wider healthcare settings (academic, community, and private hospitals and healthcare centers) are warranted.

## Introduction

Laboratory investigations are a fundamental part of diagnosing, treating, and monitoring diseases in modern medicine [1]. However, tests can be ordered inappropriately, resulting in either overutilization (ordering tests more than needed) or underutilization (omitting tests while clinically indicated) [2]. Overutilization can also occur in two ways: tests are ordered at a patient’s initial meeting or repeated after they have already been performed recently. A meta-analysis found that 20% of ordered tests were overutilized [2], while single studies (Cadamuro et al.) found that to be 70% [3].

Although laboratory investigations only represent 2.3% and 1.4% of total healthcare expenditure (HCE) in the United States and Germany, respectively [4], the downstream payment of laboratory investigations is undoubtedly expected to be larger, especially if inappropriately ordered, as overutilization can lead to unwanted sample-collection procedures, incorrect diagnoses, unnecessary additional investigations, and longer hospital stays. As such, multiple strategies and campaigns have been implemented to reduce unnecessary laboratory investigations [5].

Antinuclear antibodies (ANA) are a group of autoantibodies that are associated with and used to diagnose autoimmune connective tissue diseases, such as systemic lupus erythematosus (SLE), systemic sclerosis, primary Sjögren syndrome, mixed connective tissue disease, and idiopathic inflammatory myopathies (such as polymyositis and dermatomyositis) [6]. ANA bind the nucleic acids DNA and RNA and their associated proteins and complexes. ANA and their autoantigens form immune complexes, which get implanted into tissues to damage cells and induce cytokine production and complement activation [7].

ANA are often tested as a group, and results are reported either positive or negative with a titer and immunofluorescence-staining pattern. Additional testing is then carried out to define the specific ANA present, which can be antibodies to Sm, double-stranded DNA (dsDNA), SSA/Ro60, U1RNP, topoisomerase I, centromere protein B (CENPB), RNA polymerase III, and Jo1 [6].

ANA at a titer of ≥1:80, for the first time, have been added in 2019 to the diagnosis criteria of SLE [8]. The corresponding sensitivity and specificity of that titer are 97.8% and 74.7%, respectively [9]; ANA can be present in healthy individuals and certain populations, which can be as high as 50% [10], explaining the low specificity of such a low titer concentration.

Because testing for ANA is frequently performed unnecessarily by clinicians in patients presenting with symptoms such as widespread pain and fatigue, the American Society for Clinical Pathology and American Society for Clinical Laboratory Science recommended preserving the test for patients, with a high pre-test probability for connective tissue disease [11], as a positive result can be present in a healthy subject. Moreover, following positive results in patients with a connective tissue disease, rheumatologists often repeat ANA testing unnecessarily, and as a result, the American College of Rheumatology and British Society for Rheumatology also are against repeating ANA testing unless the disease picture has changed [12,13].

In this study, we retrospectively assess how frequently ANA testing is repeated in the adult population of Saudi Arabia (SA) and which factors are associated with and lead to inappropriate testing.

## Materials and methods

Study design, setting, and time

This was a retrospective study conducted at King Abdulaziz University Hospital (KAUH), Jeddah, Saudi Arabia (SA) from July 2023 to September 2023.

Study participants

The inclusion criteria were patients admitted to KAUH and who underwent ANA testing from 2018 to 2022. The exclusion criteria were patients who were aged less than 18 years or with incomplete data.

Data collection and sampling technique

ANA testing Current Procedural Terminology (CPT) codes (11521 for ANA profile and ANA pattern and 3854 for ANA antibodies) were used in the KAUH Laboratory database to retrospectively find patients who met our inclusion criteria. The KAUH Laboratory database retrieved 40,634 patients who had undergone 229,825 ANA tests from 2018 to 2022. Simple information regarding the demographics of each patient, the International Classification of Diseases (ICD)-coded diagnosis at the time of ordering the ANA tests, and specialties and locations of the ordering physicians of the ANA tests were also collected from the database. From the 40,634 patients (hereafter referred to as the study group), a random sample of 500 patients was selected to thoroughly search the electronic health records (EHRs) of each patient to collect the complete variables of interest for the study. Patient-related variables included demographic information about the patients, comorbidities, and the presence of connective tissue disease. ANA test-related variables included the number of ordered ANA tests, results, interval between tests, seroconversion of ANA tests (change of ANA results from positive to negative and vice versa), and order location. Variables associated with physicians included the corresponding specialties of the clinicians who ordered the ANA test for the patient.

Data handling

Nationality and residency variables were divided into “Saudi” vs. “non-Saudi” and “Jeddah” vs. “non-Jeddah,” respectively. ICD diagnosis codes extracted from the KAUH Laboratory database were categorized into their main categories using WHO’s ICD-10 online application (2019 version) [14]. Connective tissue diseases were grouped according to their organ involvement into systemic diseases (such as SLE, rheumatoid arthritis (RA), and scleroderma), organ-specific (such as myasthenia gravis, gouty arthritis, and multiple sclerosis), and vasculitides.

ANA test results and titers were provided by the KAUH Laboratory and registered in the EHRs. In the random sample and for each patient, 10 ANA tests were collected, starting from the most recent test to the oldest. If a patient had >10 ANA tests, we labeled such patients and omitted tests above the 10th ANA test from our analysis. We considered the test “positive” if the titer was ≥1:80, and “negative” if it was either written negative by the laboratory or the ANA titer was 1:40. For ANA tests with an order in the EHR that were paired with a location and ordering physician but with no results associated with them or the KAUH Laboratory asking to repeat the sample, we labeled such tests as “not reported.”

We considered an ANA test “repeated” if there was a previous ANA test preceding it with a result and order location. The proportion of seroconversion for each ANA titer (expressed in percentage) was calculated as follows: (number of changed ANA tests)/(number of changed ANA tests + number of unchanged ANA tests) × 100. While judging the repetition, we considered the act of repeating the ANA test “justified” if the patient and/or disease’s picture changed in light of new symptoms, or if the sample of the ANA test needed to be reordered again because the laboratory could not handle the previous test that was sent (e.g., because of insufficient volume and a clogged or wrong test tube).

The location of the ordered ANA tests was divided into inpatient (I), outpatient (O), and emergency (E) departments. In the study group, we divided physicians’ specialties into internal medicine, family medicine, emergency medicine, obstetrics & gynecology, surgery, ENT, and ophthalmology, among others. In the sample group, the specialties were divided into internal medicine, rheumatology, family medicine, and others.

Data analysis

The data were analyzed statistically using SPSS version 26 (IBM Corp., Armonk, NY). To test the relationship between variables, qualitative data were expressed as numbers and percentages, and the chi-squared test (χ2) was used. Quantitative data were expressed as mean and standard deviation (mean ± SD), and non-parametric variables were tested using the Mann-Whitney and Kruskal-Wallis tests. Correlation analysis was performed using Spearman’s test, and a p-value of less than 0.05 was considered statistically significant.

Ethical considerations

Ethical approval for the study (Reference No.: 192-23) was obtained from the Research Ethics Committee of KAUH, Jeddah, SA. Patient privacy was strictly protected throughout the study, and data collection and analysis were conducted with the utmost honesty and transparency.

## Results

Study group of 40,634 patients and 229,825 ANA tests

Demographics

The KAUH Laboratory database revealed 229,825 ANA tests, which were performed on 40,634 patients from 2018 to 2022. Of the total study group of 40,634 patients, the majority of the population comprised females (30,282 individuals), representing 74.5%. In contrast, males accounted for 10,351 individuals, making up 25.5% of the total population (Table 1). The mean age of the study participants was 45.14 years (SD = 0.075), with a 95% confidence interval ranging from 45.00 to 45.29.

**Table 1:** Descriptive statistics of demographic variables (N = 40,634).

Mean age	Years					
Female	44.7 ± 14.69					
Male	46.43 ± 16.18					
Both	45.14 (SD = 0.075)					
Gender/year	2018	2019	2020	2021	2022	Total
Female	75.5%	74.8%	73.7%	73.9%	74.4%	74.5%
Male	24.5%	25.2%	26.3%	26.1%	25.6%	25.5%
Nationality/year	2018	2019	2020	2021	2022	Total
Saudi Arabian	63.3%	67.8%	70.6%	74.4%	77.0%	70.1%
Other	36.7%	32.2%	29.4%	25.6%	23.0%	29.9%

The majority, accounting for 28,474 individuals (70.1%) of the study group, were of Saudi Arabian nationality, while the rest, consisting of 12,159 individuals (29.9%), encompassed various nationalities such as Yemen (7.80%), Egypt (3.21%), India (2.88%), Pakistan (2.08%), and the Philippines (1.94%), among others (Table 1). The results of the χ2 test indicated a highly significant association between nationality and year (χ² = 459.825, df = 4, p < 0.001), suggesting a substantial variation in the distribution of nationalities across the study years, especially during the years impacted by the COVID-19 pandemic.

Diagnosis at the Time of Ordering

In examining the main diagnostic categories with a total percentage greater than 3%, several noteworthy trends emerge (Table 2) (Table A1 in the Appendix provides the most common diagnoses in each category).

**Table 2:** Diagnosis descriptions by year (N = 229,825). Check Appendix Table A1, in which examples of diagnoses in each category are provided.

Diagnosis description/year	2018	2019	2020	2021	2022	Total
Certain conditions originating in the perinatal period	0.03%	0.05%	0.03%	0.04%	0.02%	0.03%
Certain infectious and parasitic diseases	1.72%	1.57%	1.26%	1.04%	1.00%	1.34%
Codes for special purposes	1.11%	1.64%	2.27%	2.47%	2.36%	1.92%
Codes for special purposes (new technologies)	0.77%	0.31%	0.35%	0.48%	0.64%	0.52%
Congenital malformations, deformations, and chromosomal abnormalities	0.38%	0.41%	0.36%	0.37%	0.32%	0.37%
Diseases of the blood and blood-forming organs and certain disorders involving the immune mechanism	2.34%	2.98%	3.82%	3.86%	2.66%	3.08%
Diseases of the circulatory system	8.17%	8.01%	8.48%	8.64%	8.76%	8.40%
Diseases of the digestive system	4.74%	4.97%	4.35%	4.63%	4.35%	4.62%
Diseases of the ear and mastoid process	1.99%	2.08%	2.07%	2.67%	2.60%	2.27%
Diseases of the eye and adnexa	2.69%	3.19%	2.81%	2.97%	3.32%	2.99%
Diseases of the genitourinary system	11.32%	7.98%	7.44%	7.57%	9.13%	8.83%
Diseases of the musculoskeletal system and connective tissue	12.34%	12.62%	12.40%	12.54%	13.21%	12.61%
Diseases of the nervous system	0.93%	0.82%	0.87%	0.98%	1.03%	0.93%
Diseases of the respiratory system	5.00%	5.40%	4.83%	3.77%	4.56%	4.72%
Diseases of the skin and subcutaneous tissue	1.78%	1.46%	1.54%	1.61%	1.65%	1.62%
Endocrine, nutritional, and metabolic diseases	6.59%	6.28%	6.45%	7.04%	7.61%	6.79%
External causes of morbidity	0.68%	0.70%	0.05%	0.05%	0.08%	0.34%
Factors influencing health status and contact with health services	15.63%	16.25%	16.31%	15.11%	11.95%	15.06%
Injury, poisoning, and certain other consequences of external causes	0.95%	1.15%	0.85%	0.89%	0.82%	0.94%
Mental, behavioral, and neurodevelopmental disorders	10.06%	9.87%	9.96%	10.74%	10.72%	10.27%
Neoplasms (tumors)	1.33%	1.34%	1.90%	1.47%	1.20%	1.43%
Place of occurrence	0.02%	0.01%	0.02%	0.02%	0.02%	0.01%
Pregnancy, childbirth, and the puerperium	2.81%	3.36%	2.71%	2.81%	2.60%	2.87%
Symptoms, signs, and abnormal clinical and laboratory findings, not elsewhere classified	6.56%	7.44%	8.72%	8.10%	9.27%	7.92%
Unspecified	0.06%	0.12%	0.14%	0.14%	0.11%	0.11%
Total	100.00%	100.00%	100.00%	100.00%	100.00%	100.00%

Factors influencing health status and contact with health services (total: 15.06%): The percentage in this category remained relatively consistent over the years, with a noticeable decrease in 2022 compared to previous years, which may reflect changes in health behavior after the COVID-19 pandemic.

Diseases of the musculoskeletal system and connective tissue (total: 12.61%): The percentage exhibited consistent fluctuations over the years, indicating potential trends that warrant further investigation.

Mental, behavioral, and neurodevelopmental disorders (total: 10.27%): The percentage remained relatively stable, with a slight decrease in 2022.

Diseases of the genitourinary system (total: 8.83%): Fluctuations in percentages, particularly in 2018 and 2022.

Symptoms, signs, and abnormal clinical and laboratory findings (total: 7.92%): The percentage increased over the years, peaking in 2022. Statistical analysis is essential to assess the significance of this trend.

Endocrine, nutritional, and metabolic diseases (total: 6.79%): The percentage increased over the years, with a noticeable rise in 2022. The last two categories have grown in the last few years. This could be related to the side effects of COVID-19 post-pandemic treatment.

Episode Type at the Time of Ordering

Among the participants who underwent ANA testing, the distribution demonstrated a predominant presence of outpatient episodes, followed by inpatient and emergency episodes (Table 3). Emergency episodes (E) were observed in 25,250 instances, representing 10.9% of the total dataset. Inpatient episodes (I) accounted for 49,696 occurrences, making up 21.4% of the total dataset. The majority of episodes fell under the outpatient category (O), with 156,872 instances comprising 67.7% of the total dataset.

**Table 3:** Episode type by year (N = 229,825). E, emergency department; I, inpatient department; O, outpatient department.

Episode type/year	2018	2019	2020	2021	2022	Total
E	10.40%	11.40%	11.60%	10.20%	11.00%	10.90%
I	22.40%	19.70%	22.70%	21.70%	20.70%	21.40%
O	67.20%	68.90%	65.60%	68.10%	68.30%	67.70%

Ordering Departments and Services of ANA Tests

The distribution of ANA tests across various departments provided insights into the healthcare landscape within the study (Table 4). The most prevalent department based on the provided data was internal medicine (Department 106), with a frequency of 112,799, which emerged as the most frequently utilized facility, constituting a substantial 49% of the total tests. Family medicine (Department 22) was also prominently featured, conducting 28,774 tests, accounting for 12.52% of the total. This department held a notable position in the distribution of ANA tests. Following closely, obstetrics & gynecology (Ob/Gyn; Department 107) conducted 21,426 tests, representing 9.2% of the total, indicating significant utilization in the study. Surgery (Department 108) performed 19,063 tests, contributing 8.2% to the overall distribution. Emergency medicine (Department 129) conducted 18,339 tests, constituting 7.9% of the total.

**Table 4:** Department by year (N = 229,825). KAUH, King Abdulaziz University Hospital.

Department/year	KAUH code	2018	2019	2020	2021	2022	Total
Internal medicine	106	47.98%	46.71%	51.15%	49.66%	50.54%	49.08%
Obstetrics & gynecology	107	9.62%	10.73%	8.92%	8.62%	8.12%	9.24%
Surgery	108	7.81%	8.70%	7.30%	8.20%	9.07%	8.22%
ENT	110	2.43%	2.95%	2.42%	2.52%	2.59%	2.58%
Others	112	0.17%	0.06%	0.12%	0.51%	0.46%	0.27%
Intensive care unit (ICU)	114	0.48%	0.45%	0.74%	0.66%	0.48%	0.55%
Anesthesia	127	1.30%	1.35%	1.17%	1.37%	0.77%	1.20%
Emergency medicine	129	7.90%	7.90%	8.29%	7.62%	7.91%	7.91%
Radiology	133	0.12%	0.18%	0.14%	0.28%	0.25%	0.19%
Surgery (Oral and maxillofacial surgery)	161	0.16%	0.20%	0.13%	0.15%	0.07%	0.14%
Family medicine	22	13.89%	13.27%	12.11%	11.48%	11.45%	12.52%
Ophthalmology	48	1.97%	1.96%	2.11%	2.83%	2.73%	2.31%
Surgery (Orthopedic services)	49	3.72%	3.25%	2.69%	2.99%	3.38%	3.24%
Pediatrics	50	0.93%	0.94%	1.25%	1.15%	0.89%	1.02%
Surgery (Urology)	51	1.52%	1.36%	1.46%	1.96%	1.28%	1.52%

Among the departments, the distribution of services revealed a diverse array of responses, with certain services standing out based on their count and percentage in the total dataset (Table A2, Appendix). Noteworthy services, each accounting for over 1% of the total responses, are as follows: medical services (Service 106, including rheumatology) of the internal medicine department emerged prominently with a count of 42,184, constituting a significant 18.2% of the total responses. Within the broader category of the internal medicine department, nephrology (Service 106.14) held a substantial presence, accounting for 14,431 instances, or 6.2% of the total. Hematology service (Service 109) was a significant contributor, with a count of 15,963, representing 6.9% of the total. General arrhythmia service (Service 140) was noteworthy, contributing 6,610 responses and constituting 2.9% of the total. Ob/Gyn service (Service 107) was notable, comprising 19,783 responses and contributing to 8.5% of the total dataset. Surgical service (Service 108) represented 5.2% of the total responses, with a count of 11,963. Emergency unit services (Service 129) stood out with a count of 18,335, making up 7.9% of the total responses. General clinics service of the family medicine department (Service 22) played a prominent role, with 17,848 instances, making up 7.7% of the total dataset. Ophthalmology service (Service 48) was notable, with a count of 5,355, representing 2.3% of the total responses. Similarly, the orthopedic surgery service (Service 49) accounted for 3.2% of the total responses, with a count of 7,512. Urology service (Service 51) was also significant, contributing 3,523 responses and representing 1.5% of the total.

Randomly selected sample of 500 patients and 998 ANA tests

Demographic Information

From the study group of 40,634 patients, 500 patients were randomly selected, of whom four were excluded because of missing data on multiple variables of interest. The final number of the random sample was 496 patients who did approximately 998 ANA tests. The mean age of the studied random sample’s participants was 47.9 ± 15.53 years. Of them, 73.6% were female, 81.9% had an SA nationality, and 81.7% were Jeddah residents, all consistent with the analysis of the study group. More than half (58.7%) of the 496 patients had comorbidities (diabetes, hypertension, cardiovascular disease, etc.) and 14.7% had connective tissue diseases; of them, 61.7% had a disease with a systemic nature (SLE, RA, scleroderma, and Sjögren-Larsson syndrome), while 34.2% had organ-specific diseases (myasthenia gravis, bilateral flexor tenosynovitis, gouty arthritis, multiple sclerosis, myopathy, osteoarthritis, psoriasis, sacroiliitis, and fibromyalgia) and 4.1% had vasculitides (Behcet disease). Of patients who had SLE (total: 23), seven (30.4%) had lupus nephritis (Table 5).

**Table 5:** Distribution of studied participants according to their demographic characteristics, comorbidity, connective tissue diseases, and SLE, as well as lupus nephritis comorbidity (N = 496). SLE, systemic lupus erythematosus.

Variable	No. (%)
Age (years) (mean ± SD)	47.9 ± 15.53
Gender	
Female	365 (73.6)
Male	131 (26.4)
Nationality	
Non-Saudi	90 (18.1)
Saudi	406 (81.9)
Residency	
Jeddah	405 (81.7)
Non-Jeddah	17 (3.4)
Not found	74 (14.9)
Comorbidities	
Yes	291 (58.7)
No	205 (41.3)
Is there a connective tissue disease present?	
No	423 (85.3)
Yes	73 (14.7)
If the answer is “Yes,” specify: (N = 73)	
Systemic	45 (61.7)
Organ-specific	25 (34.2)
Vasculitides	3 (4.1)
If the patient has SLE, does he or she also have lupus nephritis? (N = 23)	
No	16 (69.6)
Yes	7 (30.4)

ANA Tests

**Figure 1:**
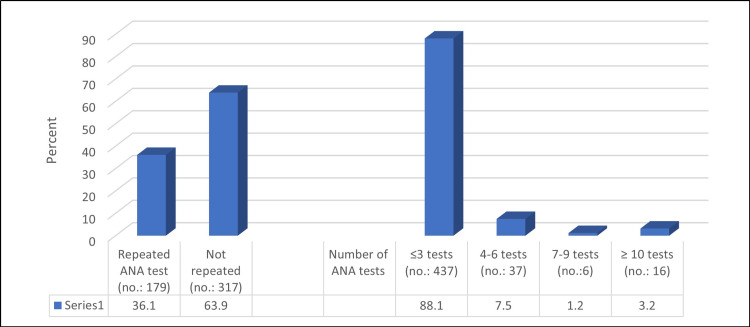
Percentage distribution of the repeated ANA tests and their numbers (groups) (N = 496). ANA, antinuclear antibodies.

The randomly selected sample of 496 patients underwent 998 tests over the years. Out of the patients, 317 (63.9%) had only one ANA test, and in total, 36.1% (179) patients had repeated ANA tests (Figure 1).

Positive results constituted 57% of the total 998 ANA test results (Table 6). When repeated (count 479 ANA tests), the interval between tests was mostly more than one year, but results remained unchanged in 67% of repeated tests (count 323 tests out of 479 repeated tests). The most common specialties to request ANA testing were internal medicine and rheumatology, representing 50% (546 ANA tests) and 21% (215 tests), respectively, of ordering specialties. Family medicine represented 8% (82 tests) of ordered ANA tests. The majority of the ANA tests were ordered from the outpatient (O) settings (78%), followed by inpatient (14%) and emergency (E) (8%) departments (Table 6).

**Table 6:** Distribution of ordered ANA tests and their result, interval, ordering physician’s specialty, and order location (N = 998). ANA, antinuclear antibodies.

Variable	No. (%)
1st–10th ANA tests	
Result of ANA test	
Negative or titer of 1:40	334 (33)
Not reported	98 (10)
Positive	566 (57)
Interval between the tests	
3 months or less	102 (21)
6 months or less	77 (16)
1 year or less	91 (19)
More than 1 year	163 (34)
Missing	47 (10)
Seroconversion among repeated tests	
From positive to negative	41 (9)
From negative to positive	25 (5)
Unchanged	323 (67)
Could not assess	90 (19)
Ordering physician’s specialty of ANA test	
Family medicine	82 (8)
Internal medicine	546 (51)
Rheumatology	215 (20)
Other	224 (21)
Location of the order	
Emergency department (ED/ER)	65 (8)
Inpatient department (wards)	105 (14)
Outpatient department (OPD)	598 (78)

Table 7 shows that, when positive, most of the titers were of mild positivity of 1:80 (22%) and 1:160 (13%). This pattern was similar to whether the patient did only one ANA test or whether his or her tests were repeated. We found that only 334 (33%) of the total ordered 998 ANA tests were negative (including those with a titer of 1:40), 98 (10%) were unreported, and 566 (57%) were positive (Table 6). The most common type of seroconversion was from positive results to negative ones, which happened to 9% (count 41 out of 479) of repeated tests. Mild-positivity (1:80-1:160) ANA tests constituted most of the change (Table 8). Of the 479 repeated ANA tests examined, only 25 (5%) ANA tests had their results changed from negative to positive. Our data revealed that ANA tests with moderate positivity (1:320-1:640) and strong positivity (≥1280) results tended not to seroconvert, as that happened to 1.7% and 0%, respectively, of reported tests.

**Table 7:** Distribution of titers of ordered ANA tests (N = 998). ANA, antinuclear antibodies.

Titer	No. (%)		
	Only 1 ANA test	≥2 (repeated) ANA tests	All ANA tests
1:40	21 (7)	54 (8)	75 (8)
1:80	83 (26)	134 (21)	217 (22)
1:160	33 (10)	96 (15)	129 (13)
1:320	13 (4)	54 (8)	67 (7)
1:640	15 (5)	30 (5)	45 (5)
1:1280	8 (3)	50 (8)	58 (6)
Negative	121 (38)	160 (25)	281 (29)
Not reported	22 (7)	74 (11)	96 (10)

**Table 8:** Distribution of seroconversion among the repeated ANA tests (N = 479). ANA, antinuclear antibodies; NA, not applicable.

	Type of change, No. (%)				
Titers	From positive to negative	From negative to positive	Unchanged	Not applicable	Proportion of seroconversion (%)
All titers combined	41 (9)	25 (5)	323 (67)	90 (19)	17
Negative or weak positivity (1:40)	0	25	105	12	19
Mild positivity (1:80-1:160)	40	0	115	16	25
Moderate positivity (1:320-1:640)	1	0	58	10	1.7
Strong positivity (>= 1280)	0	0	38	4	0
Not reported	0	0	7	48	NA

**Figure 2:**
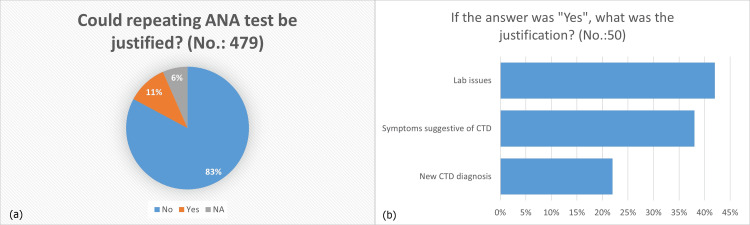
Distribution of the justification outcome of repeated ANA tests (N = 479). (A) Was there a need for ANA tests to be repeated? (B) If repeating an ANA test was reasonable, what was the justification? ANA, antinuclear antibodies; CTD, connective tissue disease.

While looking at patients’ whole pictures and correlating that with ANA test results and intervals, to assess whether repeating ANA tests more than once can be justified, we found that in 83% of repeated ANA tests (count 391 out of the 479 repeated tests), reordering ANA tests was unnecessary. In 6% of the assessed repetitions (count 31 repeated ANA tests), the data were not enough to judge this outcome. Only in 11% of the repeated ANA tests (count 50), the repetition could be justified (Figure 2A). Of the 50 instances in which we found repeating ANA tests was reasonable, 42% (count 21 repeated ANA tests) were due to laboratory-related issues, while 38% (count 19) were due to new symptoms that could be related to a yet-to-be-diagnosed connective tissue disease. Only in 22% of the instances (count 11), repeating ANA tests led 11 patients to be diagnosed with connective tissue disease for the first time, as ANA tests were reordered (Figure 2B).

Correlational Analysis of Repeated ANA Tests

Our analysis found that patients with repeated ANA tests had a significantly higher percentage of being a Jeddah resident (p < 0.05) and having no connective tissue disease (72.1% vs. 27.9%) (Table 9). Of those patients who had ≥10 ANA tests, the majority of them were females and had connective tissue disease with systemic involvement (p < 0.05) (Table 10).

**Table 9:** Relationship between repeated tests and participants’ demographics, comorbidity, connective tissue diseases, and SLE and lupus nephritis comorbidity (N = 496). SD, standard deviation; SLE, systemic lupus erythematosus. N.B.: * Kruskal–Wallis test.

Variable	Repeated test		χ^2^	p-value
	No, No. (%)	Yes, No. (%)		
Age (years) (mean ± SD)	47.79 ± 15.99	48.11 ± 14.74	0.55*	0.576
Gender				
Female	230 (72.6)	135 (75.4)	0.48	0.487
Male	87 (27.4)	44 (24.6)		
Nationality				
Non-Saudi	60 (18.9)	30 (16.8)	0.36	0.547
Saudi	257 (81.1)	149 (83.2)		
Residency				
Jeddah	245 (77.3)	160 (89.4)	14.75	0.011
Non-Jeddah	12 (3.8)	5 (2.8)		
Not found	60 (18.9)	14 (7.8)		
Comorbidities				
Yes	177 (55.8)	114 (63.7)		
No	140 (44.2)	65 (36.3)	0.29	0.088
Is there a connective tissue disease present?				
No	294 (92.7)	129 (72.1)		
Yes	23 (7.3)	50 (27.9)	38.96	<0.001
If the answer is “Yes,” specify: (N = 73)				
Systemic	9 (39.1)	36 (72)		
Organ-specific	14 (60.9)	11 (22)	11.09	0.004
Vasculitides	0 (0.0)	3 (6)		
If the patient has SLE, does he or she also have lupus nephritis? (N = 23)				
No	2 (0.6)	14 (7.8)		
Yes	0 (0.0)	7 (3.9)	32.21	<0.001

**Table 10:** Relationship between the number of ANA tests and participants’ demographics, comorbidity, connective tissue diseases, and SLE and lupus nephritis comorbidity (N = 496). ANA, antinuclear antibodies; SD, standard deviation; SLE, systemic lupus erythematosus. N.B.: * Kruskal–Wallis test.

Variable	Number of ANA tests				χ^2^	p-value
	≤3 tests (N = 437)	4–6 tests (N = 37)	7–9 tests (N = 6)	≥10 tests (N = 16)		
Age (years) (mean ± SD)	47.94 ± 15.98	49.24 ± 10.17	47.17 ± 18.6	44 ± 12.45	3*	0.611
Gender						
Female	310 (70.9)	34 (91.9)	5 (83.3)	16 (100)	13.99	0.003
Male	127 (29.2)	3 (8.1)	1 (16.7)	0 (0.0)		
Nationality						
Non-Saudi	81 (18.5)	4 (10.8)	0 (0.0)	5 (31.3)	4.56	0.207
Saudi	356 (81.5)	33 (89.2)	6 (100)	11 (68.8)		
Residency						
Jeddah	352 (80.5)	33 (89.2)	4 (66.7)	16 (100)	23.29	0.078
Non-Jeddah	14 (3.1)	3 (8.1)	0 (0.0)	0 (0.0)		
Not found	71 (17.2)	1 (2.7)	2 (33.3)	0 (0.0)		
Comorbidities						
Yes	253 (57.9)	26 (70.3)	3 (50)	9 (56.3)		
No	184 (42.1)	11 (29.7)	3 (50)	7 (43.8)	2.38	0.496
Is there a connective tissue disease present?						
No	394 (90.2)	26 (70.3)	1 (16.7)	2 (12.5)		
Yes	43 (9.8)	11 (29.7)	5 (83.3)	17 (87.5)	14.95	<0.001
If the answer is “Yes,” specify: (N = 73)						
Systemic	19 (44.2)	9 (81.8)	5 (100)	12 (85.7)		
Organ-specific	21 (48.8)	2 (18.2)	0 (0.0)	2 (14.3)	14.29	0.026
Vasculitides	3 (7)	0 (0.0)	0 (0.0)	0 (0.0)		
If the patient has SLE, does he or she also have lupus nephritis? (N = 23)						
No	4 (0.9)	4 (10.8)	2 (33.3)	6 (37.5)		
Yes	0 (0.0)	1 (2.7)	1 (16.7)	5 (31.3)	21.52	<0.001

Table 11 shows that rheumatology, out of all specialties, had a significantly higher percentage of physicians who ordered more than 10 ANA tests (p < 0.05). Lastly, we found that patients who had an organ-specific connective tissue disease had a significantly higher percentage of having ≥10 ANA tests or repeated ANA tests in general (p < 0.05) (Table 12).

**Table 11:** Relationship between ordering physician’s specialty and number of ANA tests (N = 496). ANA, antinuclear antibodies.

	Ordering physician’s specialty				χ^2^	p-value
Variable	Family medicine	Medicine	Rheumatology	Other		
	No. (%)	No. (%)	No. (%)	No. (%)	45.02	0.038
One test	35 (64.8)	175 (61.6)	12 (41.4)	95 (73.6)		
2 tests	9 (16.7)	55 (19.4)	7 (24.1)	15 (11.6)		
3 tests	3 (5.6)	23 (8.1)	3 (10.3)	5 (3.9)		
4 tests	2 (3.7)	11 (3.9)	2 (6.9)	3 (2.3)		
5 tests	2 (3.7)	5 (1.8)	1 (3.4)	2 (1.6)		
6 tests	2 (3.7)	3 (1.1)	2 (6.9)	2 (1.6)		
7 tests	0 (0.0)	2 (0.7)	0 (0.0)	0 (0.0)		
8 tests	1 (1.9)	2 (0.7)	0 (0.0)	1 (0.8)		
9 tests	0 (0.0)	0 (0.0)	1 (3.4)	0 (0.0)		
10 tests	1 (1.9)	0 (0.0)	0 (0.0)	1 (0.8)		
>10 tests	0 (0.0)	8 (2.8)	1 (3.4)	5 (3.9)		

**Table 12:** Relationship between the number of ANA tests and participants’ demographics, comorbidity, connective tissue diseases, and SLE and lupus nephritis comorbidity (N = 496). ANA, antinuclear antibodies; SLE, systemic lupus erythematosus. N.B.: * Kruskal–Wallis test.

Variable	Type of connective tissue disease, if present				χ^2^	p-value
	Systemic	Organ-specific	Vasculitides	Other		
Number of ANA tests						
1 test	3 (15.8)	4 (44.4)	0 (0.0)	170 (64.9)	17.08	<0.001
2 tests	5 (26.3)	2 (22.2)	0 (0.0)	50 (19.1)		
3 tests	2 (10.5)	0 (0.0)	1 (100)	16 (6.1)		
4 tests	1 (5.3)	1 (11.1)	0 (0.0)	13 (5)		
5 tests	0 (0.0)	0 (0.0)	0 (0.0)	6 (2.3)		
6 tests	2 (10.5)	1 (11.1)	0 (0.0)	2 (0.8)		
7 tests	0 (0.0)	0 (0.0)	0 (0.0)	1 (0.4)		
8 tests	1 (5.3)	0 (0.0)	0 (0.0)	0 (0.0)		
9 tests	1 (5.3)	0 (0.0)	0 (0.0)	0 (0.0)		
10 tests	0 (0.0)	0 (0.0)	0 (0.0)	1 (0.4)		
>10 tests	0 (0.0)	1 (11.1)	0 (0.0)	3 (1.1)		
Number of ANA tests (groups)						
≤3 tests (N = 437)	10 (52.6)	6 (66.7)	1 (100)	236 (90.1)	17.36	<0.001
4–6 tests (N = 37)	3 (15.8)	2 (22.2)	0 (0.0)	21 (8)		
7–9 tests (N = 6)	2 (10.5)	0 (0.0)	0 (0.0)	1 (0.4)		
≥10 tests (N = 16)	4 (21.1)	1 (11.1)	0 (0.0)	4 (1.5)		
ANA test repetition						
Repeated ANA test (N = 179)	3 (15.8)	4 (44.4)	0 (0.0)	170 (64.9)	24.14	<0.001
Not repeated (N = 317)	65 (31.7)	5 (55.6)	1 (100)	92 (35.1)		

## Discussion

ANA are autoantibodies that, when tested, have high sensitivity (~98%) but low specificity (~75%) against autoimmune diseases [9]. The low specificity is likely due to ANA appearing in healthy individuals and getting elevated as a part of other non-rheumatologic conditions, such as malignancies, drug exposure, and infectious diseases [15].

When, in 2012, the American Board of Internal Medicine Foundation launched the campaign *Choosing Wisely®: Next Steps in Improving Healthcare Value *to encourage delivering evidence-based medicine among specialties and reduce cost and harm associated with unnecessary and unsupported use of investigations and treatments, ANA testing appeared at the top of the five items on which rheumatologists internationally agreed their practices needed to be questioned [16,17]. Since then and all over the globe, multiple studies have been carried out to investigate the landscape of ANA testing: out of all ANA tests, how many are ordered appropriately; which specialties order most of the tests; how much unnecessary ANA tests represent of HCE; and what interventions can be used to reduce the inappropriate ordering of ANA tests [18-20].

One of the studies was done in Ontario, Canada, and involved 587,357 ANA tests that were performed on 437,966 adult patients from 2008 to 2015 [18]. The researchers found that only 21.5% of ordered ANA tests were positive, and from 587,357 ANA tests, 28% (164,913 tests) were repeated, with almost half of the repeated (49%) reordered within a year from a previous test. Family medicine and rheumatology were the most common specialties to order ANA tests, representing 61% and 11%, respectively, of requesting specialties. Yeo et al. [19] in Australia went further and assessed the trends of 23,438 ANA tests that were performed on 19,603 patients from 2013 to 2020 and found that 95.7% of repeated tests were stable and their results did not change over time.

In this study, we retrospectively investigated similar research questions and tried to expose the landscape of ANA testing in an academic hospital in Jeddah, SA. Our study group consisted of 40,634 adult patients who underwent 229,825 ANA tests from 2018 to 2022. A random sample of 496 patients, who performed 998 ANA tests, was selected to answer the research questions in depth. We found that the majority of the patients were females (74.5% in the study group vs. 73.6% in the random sample), most patients were of SA nationality (70.1% in the study group vs. 81.9% in the random sample), and the mean age, in years, was 45.14 in the study group vs. 47.9 in the sample group.

We found the most common diagnoses associated with ordering ANA tests were "Factors influencing health status and contact with health services" (15.06%) (Table A1, Appendix, provides examples), followed by "Diseases of the musculoskeletal system and connective tissue" (12.61%). "Mental, behavioral, and neurodevelopmental disorders" represented 10.27%, "Diseases of the genitourinary system" represented 8.83%, and "Symptoms, signs, and abnormal clinical and laboratory findings" represented 7.92%. "Endocrine, nutritional, and metabolic diseases" encompassed 6.79% of ANA testing-associated diagnoses.

Contrary to the above-mentioned studies [18,19], in our study, most ANA tests were often ordered by highly specialized specialists rather than family medicine doctors (in the study group, 49% of ordered ANA tests were by the internal medicine department vs. 12.52% for that by the family medicine department). The most common locations for ordering ANA tests were outpatient settings (67.7% of ordered ANA tests), followed by inpatient departments (21.4%), and emergencies came in last with 10.9% of the total ordered ANA tests. Similar trends appeared in our analysis of the random sample. This clearly reflects the current medical practice in the SA healthcare system, where there is room for improving organization and integration of the system, to avoid patients accessing highly specialized care prior to primary care evaluation [21].

Within the sample group, we found 57% of ANA tests (count 566 out of 998 tests) to be positive, with the most common titer results of mild positivity of 1:80 (22%) and 1:160 (13%). The percentage was similar whether the patient had only one ANA test or the tests were repeated. These findings are in disagreement with the previously mentioned studies, as the majority of ANA tests were found to be negative rather than positive [18,19]. Such findings likely reflect the variability in the cut-off values used to define a positive test, which needs further assessment.

Our analysis revealed that after repetition, most of the results of repeated tests (67%) do not change, and if there was a change, the majority was from positive test results to negative ones. Most seroconversion came from negative ANA tests or those with weak (titer 1:40) or mild positivity (titer 1:80-1:160). Moderate (titer 1:320-1:640) and strong (titer ≥1280) positivity ANA tests tended to not change. These patterns of seroconversion can be explained by the ups and downs of ANA in non-rheumatologic diseases, which are more common than rheumatologic diseases [15]; and our results reinforce the notion that ANA tests do not need to be repeated unless the disease picture has changed [11-13]. When examining whether repetition of ANA tests can be justified, we found that in 83% of the instances, repeating the test was not appropriate. Only in 11% of instances (count 50), repeating ANA tests was suitable, and in 42% (count 21 out of 50), that was related to laboratory or sampling issues. Of the 179 patients who got their ANA tests repeated, repeating ANA tests led 11 patients to be diagnosed with connective tissue diseases for the first time.

Lastly, we performed a correlation analysis and found that being a Jeddah resident and having no connective tissue disease were associated with a higher percentage of getting repeated ANA tests in general. On the other hand, being a female, having a systemic connective tissue disease, and the specialist being a rheumatologist were all associated with ordering more than 10 ANA tests.

Our study has limitations. First, the studied patients and examined ANA tests were retrieved from a single academic and specialized hospital, which may have affected the numbers and results of performed ANA tests, as sicker patients are often the most served patients, which contrasts community and private practices. Second, some variables related to patients, such as comorbidities, socioeconomic status, death, and diagnosis at the time of the ANA test order, were not incorporated in-depth or at all in our analysis. Third, and although small, some ANA tests have missing data, such as the results and intervals. Lastly, we only included the specialties of the ordering physicians, but many variables (such as the clinician’s demographics, level of education, graduation program/school, and whether the repeated and initial ANA tests were ordered by the same physician or not) were not assessed. Further studies involving patients from SA and across diverse healthcare settings are needed before conclusions are drawn.

## Conclusions

The study’s aim was to assess the landscape of ANA tests and the factors that lead to the inappropriate repetition of ANA tests. Some of our findings were in alignment with other studies in the literature, while others were not. But the conclusion was the same: in most cases, ANA results mostly do not change, and repeating the tests is futile.

## Appendices

Table A1

**Table 13:** The most common diagnoses that appeared in our database from the ICD-10 online application (2019 version) of the WHO (N = 229,825). ICD, International Classification of Diseases; WHO, World Health Organization.

#	ICD category's description	Top five diagnoses
I	Certain infectious and parasitic diseases	Coronavirus infection, unspecified site; gastroenteritis and colitis of unspecified origin; chronic viral hepatitis B without delta-agent; tuberculosis of other specified organs; chronic viral hepatitis C
II	Neoplasms	Malignant neoplasm of breast, unspecified part; malignant neoplasm of the central portion of the breast; malignant neoplasm of the thyroid gland; benign neoplasm of the breast; malignant neoplasm of ovary
III	Diseases of the blood and blood-forming organs and certain disorders involving the immune mechanism	Beta thalassemia; iron deficiency anemia, unspecified; sickle-cell anemia without crisis; other iron deficiency anemia; iron deficiency anemia secondary to blood loss (chronic)
IV	Endocrine, nutritional, and metabolic diseases	Hypothyroidism, unspecified; type 2 diabetes mellitus without complication; type 2 diabetes mellitus with poor control; other obesity; insulin-dependent diabetes mellitus without complications, not stated as uncontrolled
V	Mental and behavioral disorders	Generalized anxiety disorder; moderate depressive episode, not specified as arising in the postnatal period; organic depressive disorder; organic anxiety disorder; nonorganic insomnia
VI	Diseases of the nervous system	Sleep apnea, unspecified; multiple sclerosis; carpal tunnel syndrome; myasthenia gravis; localization-related (focal)(partial) idiopathic epilepsy and epileptic syndromes with seizures of localized onset, without mention of intractable epilepsy
VII	Diseases of the eye and adnexa	Blepharitis; other senile cataract; cataract, unspecified; diabetic retinopathy; other glaucoma
VIII	Diseases of the ear and mastoid process	Other peripheral vertigo; benign paroxysmal vertigo; hearing loss, unspecified; tinnitus; otitis media, unspecified
IX	Diseases of the circulatory system	Essential (primary) hypertension; congestive heart failure; heart failure, unspecified; stroke, not specified as hemorrhage or infarction; atrial fibrillation and flutter
X	Diseases of the respiratory system	Asthma, unspecified; other allergic rhinitis; acute upper respiratory infection, unspecified; acute nasopharyngitis (common cold); allergic rhinitis, unspecified
XI	Diseases of the digestive system	Other and unspecified cirrhosis of the liver; other gastritis autoimmune hepatitis; Crohn's disease, unspecified; other Crohn's disease
XII	Diseases of the skin and subcutaneous tissue	Acne vulgaris; allergic urticaria; cellulitis of lower limb; other pruritus; other atopic dermatitis
XIII	Diseases of the musculoskeletal system and connective tissue	Low back pain; systemic lupus erythematosus, unspecified; systemic lupus erythematosus with organ or system involvement; pain in joints, multiple sites; primary gonarthrosis, bilateral
XIV	Diseases of the genitourinary system	Other chronic renal failure; end-stage renal disease; urinary tract infection, site not specified; female infertility, unspecified; calculus of kidney
XV	Pregnancy, childbirth, and the puerperium	Duration of pregnancy 5-13 completed weeks; duration of pregnancy 26-33 completed weeks; single delivery by cesarean section; duration of pregnancy 20-25 completed weeks; duration of pregnancy 14-19 completed weeks
XVI	Certain conditions originating in the perinatal period	None
XVII	Congenital malformations, deformations, and chromosomal abnormalities	None
XVIII	Symptoms, signs, and abnormal clinical and laboratory findings, not elsewhere classified	Other and unspecified abdominal pain; headache; pain, unspecified; malaise and fatigue; cough
XIX	Injury, poisoning, and certain other consequences of external causes	Anticoagulants; allergy, unspecified; sprain and strain of other and unspecified parts of the knee; sprain and strain of ankle, part unspecified; injury of the tendon of the rotator cuff of the shoulder
XX	External causes of morbidity and mortality	Anticoagulants causing adverse effects in therapeutic use; other and unspecified bacterial vaccines causing adverse effects in therapeutic use; other specified vaccines and biological substances causing adverse effects in therapeutic use; health service area; endotracheal tube wrongly placed during anesthetic procedure
XXI	Factors influencing health status and contact with health services	Laboratory examination; pharmacotherapy session for neoplasm; gynecological examination (general; routine); general medical examination; other waiting period for investigation and treatment
XXII	Codes for special purposes	Health services; tennis; motorcycling; other specified activity; unspecified activity; other specified motorsport

Table A2

**Table 14:** Service by year (N = 229,825). ENT: ear, nose, and throat surgery; OBS: obstetrics; GYNE: gynecology. * In King Abdulaziz University Hospital (KAUH), most rheumatologists have duties in the Clinical Teaching Units (CTUs), which are part of the Medical Services of the Internal Medicine Department. As such, the KAUH database registered them as “Medical Services" doctors and not “rheumatologists,” hence their representation is small. While collecting the data for the sample group of 500 patients, we were able to separate them. In the sample group, rheumatologists ordered more ANA tests than family medicine doctors.

Main service	Sub service	Sub service description	2018	2019	2020	2021	2022	Total
Anesthesia	127		1.30%	1.30%	1.20%	1.40%	0.80%	1.20%
Emergency medicine	129		7.90%	7.90%	8.30%	7.60%	7.90%	7.90%
ENT	110		2.40%	2.90%	2.40%	2.50%	2.60%	2.50%
Family medicine	22		9.70%	8.40%	8.00%	8.60%	9.50%	8.90%
Internal medicine	100	CARDIOLOGY (HEART FAILURE)	0.90%	1.00%	1.20%	1.00%	0.90%	1.00%
	106	MEDICAL SERVICES	17.60%	19.30%	19.70%	17.50%	17.20%	18.20%
	106.1	CARDIOLOGY SERVICES	2.20%	1.80%	1.90%	2.00%	1.80%	2.00%
	106.12	GASTROENTEROLOGY	1.40%	1.60%	1.40%	1.60%	1.40%	1.50%
	106.13	HEPATOLOGY	1.10%	0.90%	1.00%	1.00%	0.80%	1.00%
	106.14	NEPHROLOGY	8.60%	4.50%	5.70%	4.70%	7.20%	6.20%
	106.16	PSYCHIATRIC	0.70%	0.60%	0.70%	0.80%	0.80%	0.70%
	106.2	ADULT INFECTIOUS DISEASE	0.20%	0.30%	0.20%	0.20%	0.10%	0.20%
	106.21	OBS/MED & GESTATIONAL DIABETES	0.20%	0.20%	0.20%	0.30%	0.20%	0.20%
	106.22	GASTROENTEROLOGY AND HEPATOLOGY	1.00%	1.10%	0.80%	0.90%	0.90%	0.90%
	106.3	DERMATOLOGY SERVICES	2.10%	1.60%	1.70%	1.90%	1.70%	1.80%
	106.5	NEUROLOGY SERVICES	2.50%	2.60%	2.70%	3.20%	3.20%	2.80%
	108.11	RESPIRATORY	2.30%	2.60%	2.50%	2.00%	2.40%	2.40%
	109	HEMATOLOGY SERVICES	5.60%	6.00%	7.20%	8.00%	8.00%	6.90%
	114	INTENSIVE CARE UNIT SERVICE	0.50%	0.50%	0.70%	0.70%	0.50%	0.60%
	138	ONCOLOGY SERVICES	1.50%	1.90%	2.70%	2.50%	1.40%	2.00%
	140	GENERAL ARRHYTHMIA	3.40%	3.80%	3.30%	2.20%	1.50%	2.90%
	150	GERIATRIC MEDICINE SERVICES	0.90%	1.30%	1.30%	1.20%	1.00%	1.10%
	170	ARRHYTHMIA SERVICES	0.10%	0.10%	0.10%	0.10%	0.20%	0.10%
	50.3	ENDOCRINOLOGY	0.00%	0.20%	0.60%	1.00%	1.10%	0.60%
	106.19	RHEUMATOLOGY SERVICE*	0.10%	0.10%	0.50%	0.70%	0.70%	0.40%
Obstetrics & gynecology	107	OBS/GYNE	8.80%	9.90%	8.40%	8.00%	7.50%	8.50%
	110.6	OBSTETRIC SERVICE	0.70%	0.80%	0.50%	0.50%	0.50%	0.60%
Ophthalmology	48		2.00%	2.00%	2.10%	2.80%	2.70%	2.30%
Others	112	PHARMACY SERVICE	0.10%	0.00%	0.00%	0.30%	0.30%	0.10%
	130	LABORATORY	0.10%	0.10%	0.10%	0.20%	0.10%	0.10%
	160	AUDIOLOGY SERVICES	0.00%	0.00%	0.00%	0.00%	0.00%	0.00%
Pediatrics	200	PEDIATRIC INFECTIOUS DISEASE	0.00%	0.00%	0.00%	0.00%	0.00%	0.00%
	50	PEDIATRIC SERVICES	0.80%	0.80%	1.10%	0.90%	0.60%	0.90%
Radiology	133		0.10%	0.20%	0.20%	0.30%	0.20%	0.20%
Surgery	107.5	PLASTIC SURGERY	0.50%	0.40%	0.30%	0.50%	0.60%	0.50%
	107.6	VASCULAR SURGERY	0.80%	0.70%	0.70%	0.70%	1.00%	0.80%
	107.7	PEDIATRIC SURGERY	0.10%	0.10%	0.00%	0.10%	0.00%	0.10%
	108	SURGICAL SERVICES	5.00%	5.50%	4.40%	5.30%	5.50%	5.20%
	108.1	NEUROSURGERY	1.10%	1.30%	1.20%	1.10%	1.30%	1.20%
	108.10	CARDIOSURGERY	0.30%	0.20%	0.30%	0.20%	0.10%	0.20%
	145	THORACIC SURGERY SERVICES	0.20%	0.30%	0.40%	0.30%	0.50%	0.30%
	161.1	ORAL & MAXILLOFACIAL SURGERY	0.20%	0.20%	0.10%	0.10%	0.10%	0.10%
	49	ORTHOPEDIC SERVICES	3.70%	3.20%	2.70%	3.00%	3.40%	3.20%
	51	UROLOGY SERVICES	1.50%	1.40%	1.50%	2.00%	1.30%	1.50%
